# Designing and Constructing a Novel Artificial Pathway for Malonic Acid Production Biologically

**DOI:** 10.3389/fbioe.2021.820507

**Published:** 2022-01-19

**Authors:** Shuying Gu, Zhen Zhao, Yonghong Yao, Jingen Li, Chaoguang Tian

**Affiliations:** ^1^ Key Laboratory of Systems Microbial Biotechnology, Tianjin Institute of Industrial Biotechnology, Chinese Academy of Sciences, Tianjin, China; ^2^ National Technology Innovation Center of Synthetic Biology, Tianjin, China; ^3^ University of Chinese Academy of Sciences, Beijing, China

**Keywords:** malonic acid, *a*-keto decarboxylase, oxaloacetic acid, metabolic engineering, *Myceliophthora thermophila*

## Abstract

Malonic acid is used as a common component of many products and processes in the pharmaceutical and cosmetic industries. Here, we designed a novel artificial synthetic pathway of malonic acid, in which oxaloacetate, an intermediate of cytoplasmic reductive tricarboxylic acid (rTCA) pathway, is converted to malonic semialdehyde and then to malonic acid, sequentially catalyzed by *a*-keto decarboxylase and malonic semialdehyde dehydrogenase. After the systematic screening, we discovered the enzyme oxaloacetate decarboxylase Mdc, catalyzing the first step of the artificially designed pathway *in vitro*. Then, this synthetic pathway was functionally constructed in cellulolytic thermophilic fungus *Myceliophthora thermophila*. After enhancement of glucose uptake, the titer of malonic acid achieved 42.5 mg/L. This study presents a novel biological pathway for producing malonic acid from renewable resources in the future.

## Introduction

Malonic acid, formally known as propanedioic acid, is widely used in manufacturing processes, such as the petrochemical, pharmaceutical, and cosmetic industries. Traditionally, malonic acid is produced from fossil resources *via* petrochemical processes. The byproduct sodium cyanide is dangerous and unfriendly to the environment (Hildbrand and Pollak, 2012; Werpy et al., 2004; Klikar et al., 2017). With increasing concerns on energy and environmental problems, the production of malonic acid by microbial fermentation *via* bioconversion of renewable feedstock has generated considerable interest worldwide. However, due to a lack of knowledge about the malonic acid synthesis pathway, there is little progress on the biological production of malonic acid.

Recent advances in synthetic biology and computational biology have enabled designing novel and specific metabolic pathways for desired chemicals (Atsumi et al., 2008). However, importing and overexpressing non-native pathways in microbes may lead to metabolic imbalance, resulting in none or low titer of target products. It is critical to design artificial synthetic pathways that are compatible with the host. At present, two non-natural metabolic pathways have been sought and introduced into microbes, using malonic semialdehyde (MSA) or malonyl-CoA as the precursor (Table 1) (Song et al., 2016; Dietrich et al., 2017; Chae et al., 2020). The first one uses MSA from the deamination reaction of *β*-alanine as the precursor. In *Escherichia coli*, *β*-alanine pyruvate transaminase from *Pseudomonas aeruginosa* was overexpressed to convert *β*-alanine to produce MSA. Then, succinate semialdehyde dehydrogenase encoded by *E. coli yneI* was used to catalyze the reaction of MSA to malic acid, and the titer of malonic acid reached 3.60 g/L by fed-batch fermentation (Song et al., 2016). Another pathway is dependent on the fatty acid synthesis pathway, using malonyl-CoA as the precursor. Matthew et al. reported that acyl-CoA hydrolase encoded by *ehd3* from *Saccharomyces cerevisiae* can hydrolyze malonyl-CoA to malonic acid. During overexpression of *EHD3* in *S. cerevisiae*, *Pichia kudriavzevii*, and *E. coli*, malonic acid in the cultures was detectable. After improvement of binding affinity of acyl-CoA hydrolase to malonyl-CoA by protein engineering, production of malonic acid was increased to 0.0823 g/L by engineered *E. coli* (Dietrich et al., 2017). Both current synthetic pathways of malonic acid require a number of enzymes to convert glucose to target acid and are complex, which might lead to a low titer of malonic acid. So more research is needed to improve the production of this dicarboxylic acid, including novel biosynthetic pathway design, key enzymatic engineering, and even alternative hosts.

**TABLE 1:** Summary of microbial production of malonic acid from glucose.

Microbe	Precursor	Description	Titer	Fermentation	Refs
*Escherichia coli*	β-Alanine	+*ppc*, *aspA*, and *yneI* from *E. coli*; +*panD* from *Corynebacterium glutamicum*; +*pa4123* from *Pseudomonas aeruginosa*; Δ*ydfG*	0.45 g/L	Fed batch	Song et al. (2016)
			3.6 g/L	Shake flask	
*E. coli*	Acyl-CoA	+*ehd3* mutant from *Saccharomyces cerevisiae*	82.3 mg/L	Shake flask	Dietrich et al. (2017)
*Pichia kudriavzevii*	Acyl-CoA	+*ehd3* mutant from *S. cerevisiae*; +*Anmae1* from *Aspergillus niger*	76 mg/L	Shake flask	Dietrich et al. (2017)
*Myceliophthora thermophila*	Oxaloacetate	+*mdc* from *Ogataea parapolymorpha*; +*yneI* from *E. coli*; +*glt-1* from *Neurospora crassa*	42.5 mg/L	Shake flask	This study

Note. “+” represents overexpression of target gene; “Δ” represents disruption of target gene.

Filamentous fungi are classical predominant microorganisms for organic acid production, such as citric acid, malic acid, and lactic acid (Dave and Punekar, 2015; Kirimura et al., 2016; Li et al., 2020a). The thermophilic filamentous fungus *Myceliophthora thermophila* (synonym: *Thermothelomyces thermophilus*), possessing the ability to efficiently degrade plant biomass, represents a potential library of new industrial applications, including producing thermo-tolerant and efficient cellulolytic enzymes and synthesizing biochemicals and biofuels directly from plant biomass (Berka et al., 2011; Yang et al., 2015; Singh, 2016). *M. thermophila* has been employed to synthesize bio-based products, such as malic acid, fumaric acid, and bioethanol, through metabolic engineering using CRISPR/CAS9 system (Liu et al., 2017; Gu et al., 2018; Li et al., 2020a; Li et al., 2020b). In this study, we designed a novel synthetic pathway of malonic acid and screened the key enzyme, oxaloacetate decarboxylase, *in vitro*. In this pathway, oxaloacetic acid (OAA) can be converted to MSA and then to malonic acid and sequentially catalyzed by *a*-keto decarboxylase and MSA dehydrogenase. Moreover, this artificial synthetic pathway was functionally constructed in *M. thermophila* to produce malonic acid from glucose.

## Materials and Methods

### Strains and Culture Conditions

*M. thermophila* ATCC 42464 was obtained from the American Type Culture Collection (ATCC). The wild-type strain and its mutants were grown on Vogel’s minimal medium supplemented with 2% glucose (MM medium) at 35°C for approximately 12 days to obtain mature conidia, and antibiotics were added when needed for transformant screening.

*E. coli* DH5α and *E. coli* BL21 (DE3) were cultivated in Luria–Bertani medium supplemented with 100 μg/ml of ampicillin for plasmid selection.

### Vector Construction

For overexpressing target genes in *E. coli*, the codon-optimized genes encoding oxaloacetate decarboxylase and MSA dehydrogenase were synthesized by GENEWIZ (Suzhou, China) and inserted between *Nde*I and *Xho*I of the plasmid pET-21a(+).

For the construction of plasmids overexpressing target genes in *M. thermophila*, codon-optimized *mdc* (XP_013934857.1) from *Ogataea parapolymorpha*, under control of the strong constitutive promoter of P*ap* amplified from the plasmid pPk2, was inserted between the *Bgl*II and *Bam*HI sites of pAN52-PtrpC-Bar-PMtgpdA to generate the vector pAN52-Pap-mdc, using the NEB Gibson Assembly Kit. Similarly, the PCR fragments of the strong constitutive promoter of *pgk* (MYCTH_2316240) from *M. thermophila* and the opening reading fragment of *yneI* from *E*. *coli* were inserted between the *Bgl*II and *Bam*HI sites of pAN52-PtrpC-bar-PMtgpdA to generate the plasmid pAN52-PtrpC-bar-PMtpgk-yneI. Under control of the strong constitutive promoter of *pgk* (MYCTH_2316240) of *M. thermophila*, *Anmae1* encoding the C4-dicarboxylate transporter from *Aspergillus niger* were inserted between the *Bgl*II and *Bam*HI sites of pAN52-PtrpC-neo-PMtpgk to form the vector pAN52-PtrpC-neo-PMtpgk-Anmae1. The PCR fragments of glucose transporter-encoding genes *glt-1* from *Neurospora crassa* were inserted between the *Spe*1 and *Bam*HI sites of pAN52-PtrpC-bar-PMtgpdA to generate the plasmid pAN52-PtrpC-bar-PMtgpdA-Anmae1.

All vectors were constructed using *E. coli* DH5α. The target genes cloned into shuttle vectors were sequenced to verify the authenticity of the plasmid construction. All the primers used for plasmid construction are listed in Supplementary Table S1.

### Recombinant Proteins Expression and Purification

*E. coli* BL21 (DE3) strains harboring expression plasmids were grown at 37°C in 100 ml of LB media supplemented with 100 μg/ml of ampicillin to an optical density OD_600_ of 0.4–0.5. Then, 0.4 mM of isopropyl-β-d-thiogalactopyranoside (IPTG) was supplemented, and the cells were cultivated at 16°C for 19 h. Cells were harvested by centrifugation at 4°C, resuspended in 50 mM of Tris-HCl (pH 7.5) buffer, and crushed with an ultrasound crusher. After centrifugation, the supernatant was collected and loaded onto a Ni^2+^-NTA-agarose column pre-equilibrated with binding buffer (50 mM of TAE (Tris-acetate-EDTA) buffer, 300 mM of NaCl, and 20 mM of imidazole, pH 7.5). The retained proteins were recovered with elution buffer (20 mM of Na_2_HPO_4_–NaH_2_PO_4_, pH 7.4, 500 mM of NaCl, and 500 mM of imidazole). In order to eliminate salt and imidazole, the eluted fraction was filtered by an ultrafiltration centrifugal tube. The purified protein is stored at −20°C. The purity of the enzymes was analyzed by sodium dodecyl sulfate–polyacrylamide gel electrophoresis (SDS-PAGE), and protein concentration in supernatants was measured using a Bio-Rad protein assay kit based on absorbance at 590 nm, using bovine serum albumin as the standard.

### *In Vitro* Enzyme Reaction

To synthesize malonic acid from OAA *in vitro*, the reaction mixture (2 ml) containing 50 mM (pH 7.5) of Tris-HCl, 1 mM of NAD^+^, 1 mM of MgSO_4,_ 1 mM of thiamine pyrophosphate (TPP), excessive purified YneI (approximately 40 μl), and 100 μl of purified Mdc was used. The assay was started by the addition of 1 mM of OAA and was immediately monitored at 340 nm for 5 min. Subsequently, the reactants were analyzed by gas chromatography/mass spectrometry (GC/MS).

### Gas Chromatography–Mass Spectrometry Assay

Samples (reaction products and malonic acid standard) were dried using a CentriVap vacuum concentrator. Then, the dried samples were dissolved in 20 μl of pyridine solution containing 20 mg/ml of methoxyamine hydrochloride and incubated for 90 min at 30°C. After that, 50 μl of the MSTFA reagent (containing 1% TMCS, v/v) was added to the sample aliquots, mixed well, and then incubated for 1 h at 37°C. The mixture was assayed by GC/MS as described previously (Mao et al., 2021).

### Analytical Method of Sugar

Residual glucose in the culture was determined by high-performance liquid chromatography (HPLC) equipped with a Waters (Milford, MA, USA) 2414 refractive index detector and an Aminex HPX-87H column (Bio-Rad Laboratories, Hercules, CA, USA) at 35°C. H_2_SO_4_ measuring 5 mM was employed as the mobile phase with a constant flow rate of 0.5 ml/min. Data analysis was performed using the Waters e2695 separation module.

### Transformation of *Myceliophthora* Protoplasts

Polyethylene glycol-mediated transformation of *M. thermophila* protoplasts was performed as described previously (Liu et al., 2017). For gene overexpression, 10 µg of linearized plasmid was co-transformed into *M. thermophila* protoplasts, and an antibody was employed for transformant selection on a plate as needed. The putative transformants were selected with antibodies and followed by sequential identification *via* PCR with paired primers (Supplementary Table S1).

### Malonate-Production Medium

Shaken-flask cultivation was performed with 50 ml of medium inoculated with mature spores at a final concentration of 2.5 × 10^5^ spores/ml in 250-ml Erlenmeyer flasks. The culture was incubated at 45°C with shaking at 150 rpm, and the sample (1 ml) was taken at different intervals. Each liter of the cultivation medium contained 20 g of glucose, 0.15 g of KH_2_PO_4_, 0.15 g of K_2_HPO_4_, 0.1 g of MgSO_4_·7H_2_O, 0.1 g of CaCl_2_·2H_2_O, 8 g of Bacto peptone, 1 ml of biotin (0.1 g/L), and 1 ml of trace elements of Vogel’s salt and was sterilized by autoclaving. Subsequently, sterilized CaCO_3_ was used as a neutralizing agent at a final concentration of 20 g/L to keep the pH at approximately 6.0.

## Results and Discussion

### Design of an Artificial Biosynthetic Pathway of Malonic Acid

**FIGURE 1:**
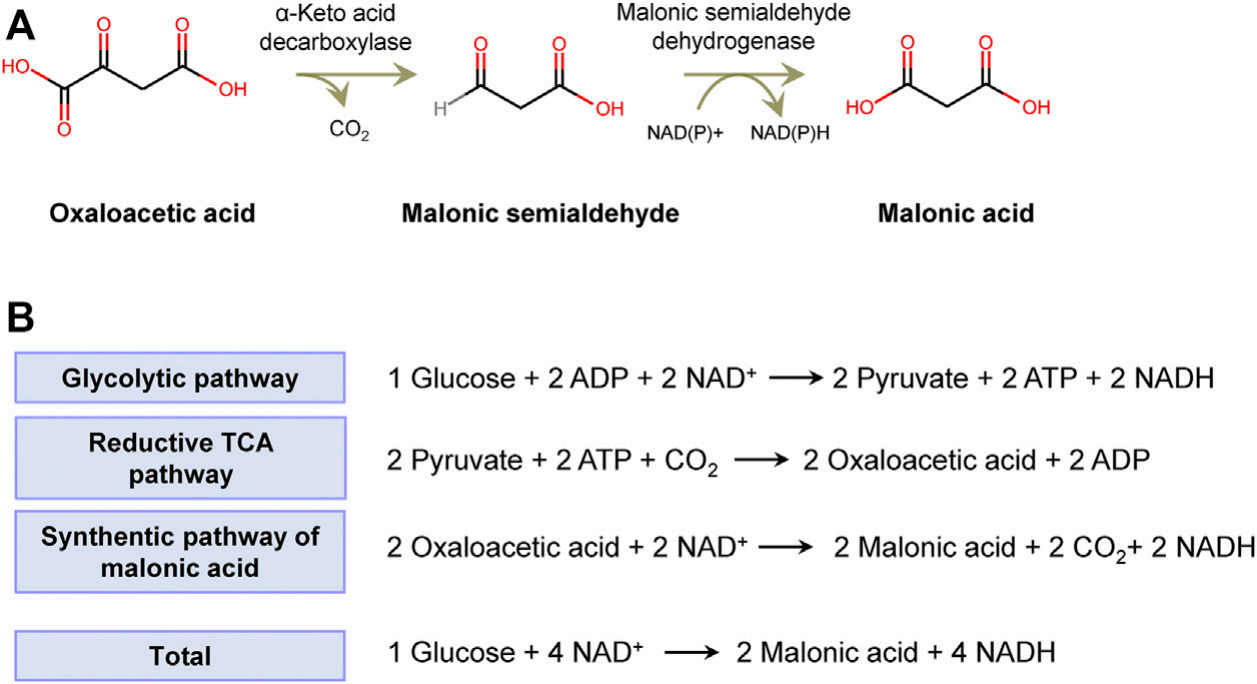
The artificial synthetic pathway of malonic acid. **(A)** Oxaloacetic acid is converted to malonic semialdehyde by *a*-keto decarboxylase and then malonic acid by malonic semialdehyde dehydrogenase. **(B)** The theoretical maximum yield of malonic acid from glucose.

A novel artificial biosynthetic route was devised to produce malonic acid with OAA as the substrate. In this route, OAA is converted to MSA by *a*-keto decarboxylase (Kdc) and then to malonic acid (Figure 1A). Kdc is the crucial enzyme step required to divert flux from OAA to malonic acid, which is widespread in plants, yeasts, and fungi. Kdc has been widely used in the non-native synthetic pathway of biofuels, in which Kdc converted 2-keto acids from amino acid biosynthesis pathways to aldehyde and then catalyzed by dehydrogenase to produce alcohols, including 1-propanol, 1-butanol, and isobutanol (Atsumi et al., 2008). Substantial numbers of Kdc have been identified and characterized. Moreover, the protein engineering and method of high-throughput screening have been developed to improve the thermostability, substrate specificity, and activity of Kdc. Therefore, it was possible to find a Kdc to catalyze OAA to MSA. In addition, another key enzyme MSA dehydrogenase has been screened, and succinate semialdehyde dehydrogenase encoded by *yneI* from *E*. *coli* has been used for catalyzing MSA to malonic acid (Fuhrer et al., 2007; Sullivan and Holyoak, 2007; Zheng et al., 2013; Song et al., 2016). Therefore, the novel pathway for synthesizing malonic acid was theoretically feasible.

In microbes, OAA is the intermediate of the cytoplasmic reductive tricarboxylic acid (rTCA) pathway and mitochondrial TCA cycle. Assuming that glucose is channeled to OAA *via* the glycolysis pathway and rTCA pathway in turn, 1 mol of glucose produces 2 mol of OAA and 2 mol of NADH, accompanied by fixation of 2 mol of CO_2_ *via* carboxylation of pyruvate catalyzed by pyruvate carboxylase. The formed 2 mol of OAA can be converted to 2 mol of malonic acid and 2 mol of NADH. In total, 1 mol of glucose produces 2 mol of malonic acid and 4 mol of NADH, by which it achieves the theoretical maximum yield of 2 mol/mol of glucose (Figure 1B). The net NADH can be used for the synthesis of cell compounds and energy metabolites.

### Synthesis of Malonic Acid by Artificial Synthetic Route *In Vitro*

**FIGURE 2:**
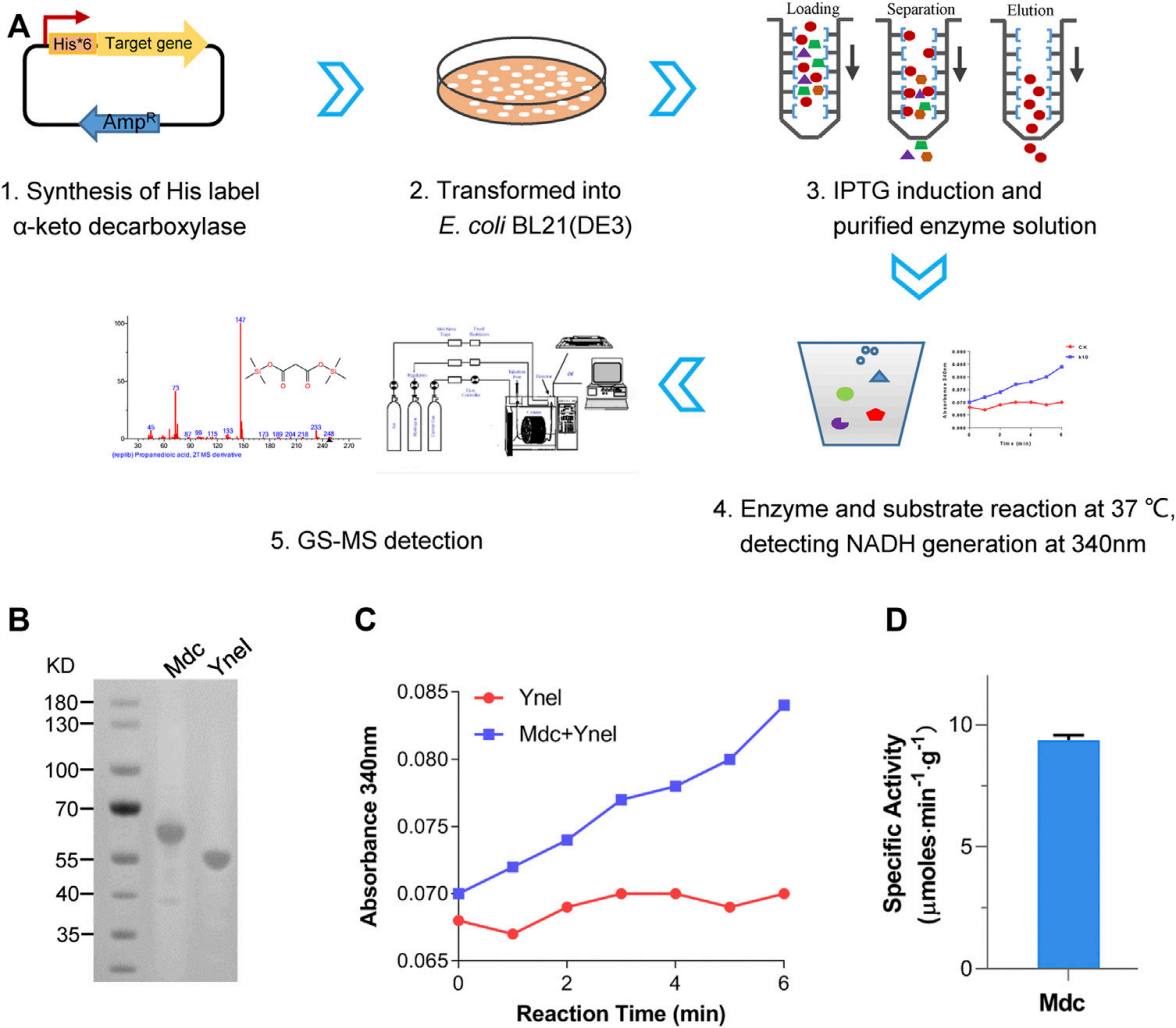
The screening of Kdc using multi-enzyme system *in vitro*. **(A)** Summary of screening *a*-keto decarboxylase. **(B)** Sodium dodecyl sulfate–polyacrylamide gel electrophoresis (SDS-PAGE) of purified Mdc (oxaloacetate decarboxylase) and YneI (malonic semialdehyde dehydrogenase). Lane M, molecular weight marker; Lane 1, elution fraction containing purified Mdc (predicted molecular mass of the monomer, 62.9 kDa); and Lane 2, elution fraction containing purified YneI (predicted molecular mass of the monomer, 50 kDa). **(C)** Time course of absorbance increase of NADH-coupled assay at 340 nm. Red: the reaction mixture only containing YneI. Blue: the reaction mixture containing Mdc and YneI. **(D)** Specific activity of purified single Mdc.

**FIGURE 3:**
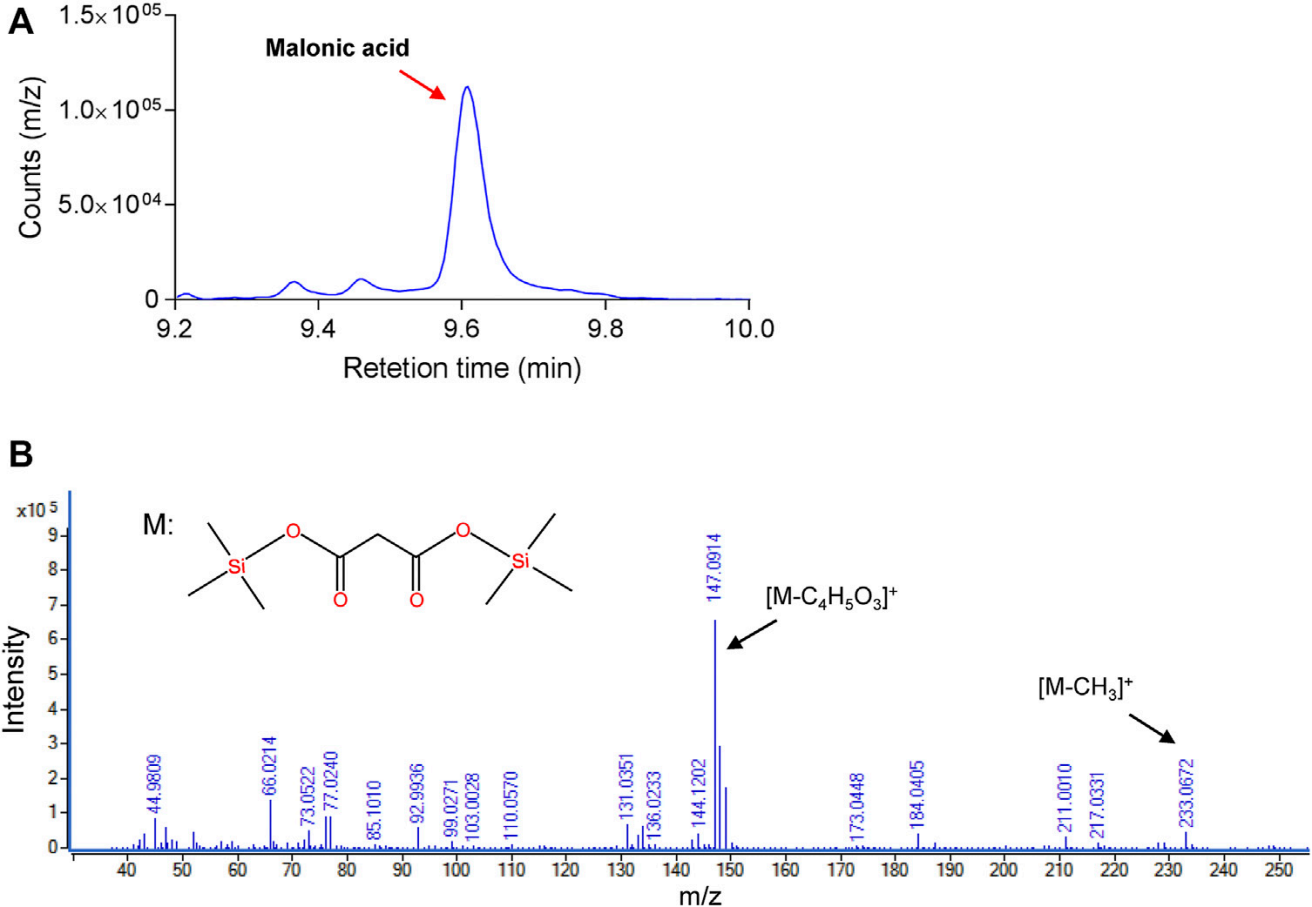
Identification of malonic acid in multi-enzyme reaction by gas chromatography/mass spectrometry (GC/MS). **(A)** GC assay of malonic acid in enzyme reaction *in vitro*. **(B)** MS spectra for synthesized malonic acid.

TPP-dependent keto decarboxylase (Kdc) is a critical enzyme in malonic acid production, and some members have been used for the production of keto acid-derived alcohols (Iding et al., 1998). During the reaction of decarboxylation of OAA, *β*-carboxylate is easier to be replaced by hydrogen atom than *a*-carboxylate, which might result in difficulty of mining the highly active enzyme (Sullivan and Holyoak, 2007). To test the capability of using OAA as a substrate, five Kdc candidates, including Thi3, Aro10 from *S. cerevisiae* (Vuralhan et al., 2003), Pdc from *Clostridium acetobutylicum* (Iding et al., 1998), Kivd from *Lactococcus lactis* (Plaza et al., 2004), and Mdc from *O. parapolymorpha*, together with succinate semialdehyde dehydrogenase-encoding YneI from *E. coli*, were heterologously overexpressed in *E. coli* BL21 (DE3) as an N-terminal His*6-tagged protein. After induction of protein expression, the target proteins were purified and characterized. The NADH-coupled enzyme assay was chosen for the characterization of these purified Kdc enzymes (Figures 2A,B). As shown in Figure 2C, the only reaction mixture containing Mdc and excessive YneI exhibited increased absorbance at 340 nm. GC/MS analysis further revealed that purified Mdc and YneI catalyzed OAA to produce malonic acid (Figure 3), and the concentration of malonic acid was up to 8.1 mg/L. These data indicated that Mdc had the ability to catalyze OAA to MSA and the specific activity achieved 9.37 μmol/min/g (Figure 2D). *In vitro* multi-enzyme system has emerged as a promising bio-manufacturing platform for producing desired products (Sun et al., 2019). The artificial biosynthetic pathway, including only two enzymes, may be feasible to produce malonic acid *in vitro* in the future.

In order to obtain oxaloacetate decarboxylase with high activity, 40 orthologs of Mdc were further chosen from the National Center for Biotechnology Information (NCBI) database by protein Blast with the protein sequence of Mdc as the query and tested for its activity of oxaloacetate decarboxylase (Supplementary Table S2). Disappointingly, none of these 40 members possessed the ability to catalyze OAA to MSA.

### The Artificial Malonate Biosynthetic Pathway Can Function in *Myceliophthora thermophila*

**FIGURE 4:**
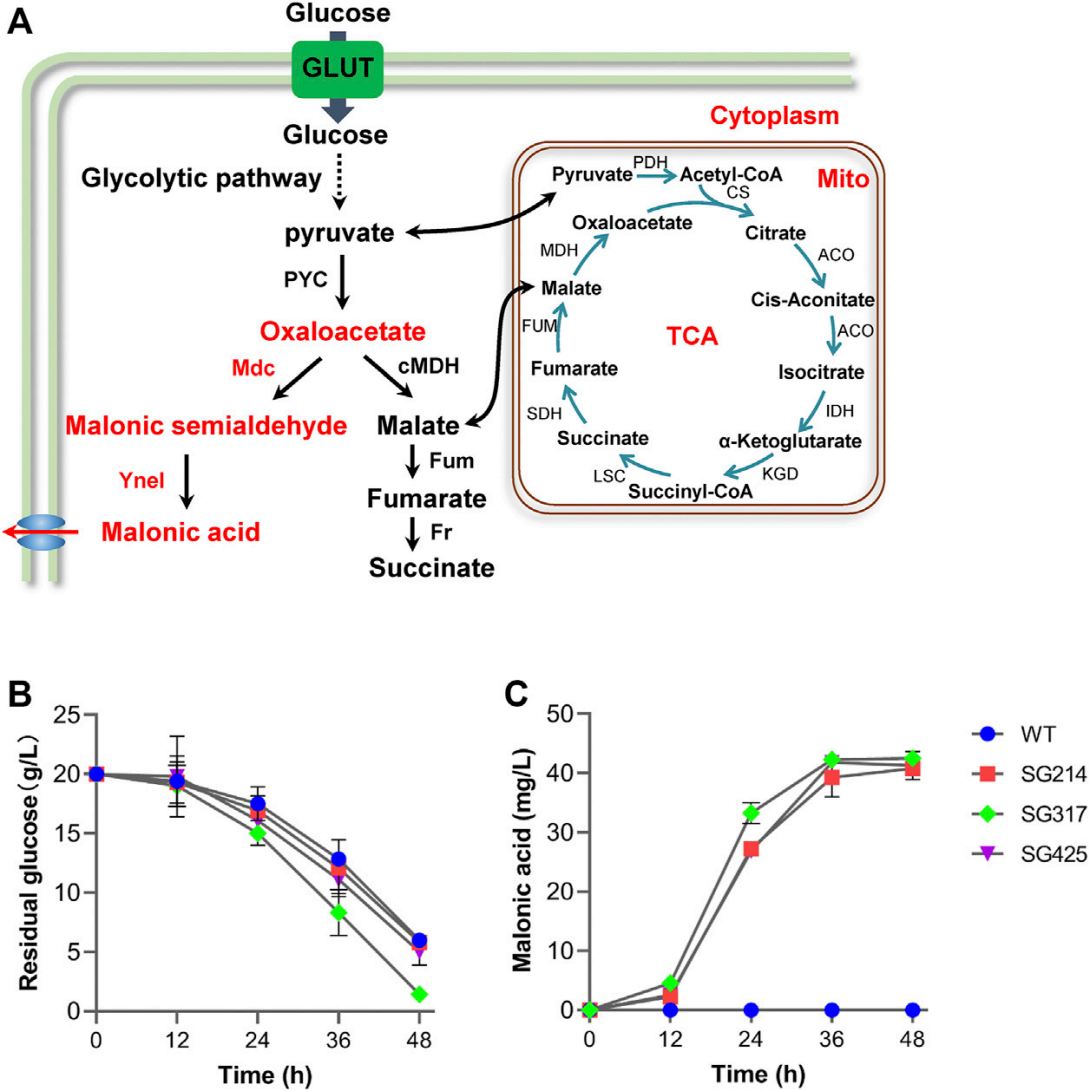
Introduction of malonic acid synthesis pathway in *Myceliophthora thermophila*. **(A)** Overall scheme of malonic acid synthesis in *M. thermophila*. Time course of glucose consumption **(B)** and malonic acid production **(C)** by *M. thermophila* mutants grown on glucose medium. WT, *M. thermophila* wild-type strain; strain SG214 containing *mdc* and *yneI*; Strain SG317 containing *mdc*, *yneI*, and *glt-1*; Strain SG425 containing *mdc*, *yneI*, and *Anmae1*; PEP, phosphoenolpyruvate; GLUT, glucose transporter; PYC, pyruvate carboxylase; cMDH, cytoplasm malate dehydrogenase; Fum, fumarase; Fr, fumarate reductase; TCA, tricarboxylic acid cycle; PDH, pyruvate dehydrogenase; CS, citrate synthase; ACO, aconitase; IDH, isocitrate dehydrogenase; KGD, а-ketoglutarate dehydrogenase complex; LSC, succinyl-CoA synthetase; SDH, succinate dehydrogenase; MDH, mitochondrial malate dehydrogenase.

Thermophilic fungus *M. thermophila* has been engineered to produce C4-dicarboxylic acids, malic acid, and fumaric acid, by cytoplasmic rTCA pathway (Gu et al., 2018; Li et al., 2020a), which can provide precursor OAA for producing malonic acid. Therefore, in order to test whether this artificial malonic pathway can function *in vivo*, both *mdc* and *yneI* were integrated into the genome of *M. thermophila* wild-type strain, under the control of the strong constitutive promoters of *ap* (transcriptional enhancer factor) and *pgk*, respectively (Figure 4A). After confirmation *via* PCR analysis, the engineered strains were tested for the production of malonic acid with glucose as the carbon source. Malonic acid in the culture of the strain of SG214 was detected by GC/MS assay, and the titer achieved 40.8 mg/L (Figure 4B), indicating that the artificial malonate synthetic pathway we designed can work functionally *in vivo*. It presented a promising approach to produce malonic acid direct from renewable plant cell biomass in this cellulolytic thermophilic fungus in the future.

### No Obvious Improvement was Obtained by Overexpression of Glucose Transporter and a Predicted Exporter of Malonic Acid

Rapid substrate supply was recognized as one strategy to maintain a high flux of reaction, which is a prerequisite for efficient cell factory production of biochemicals. Glucose transporter gene *glt-1* from *N. crassa* has been systematically characterized and used for improving the production of commodity chemicals, such as malic acid and ethanol (Chen et al., 2017; Wang et al., 2017; Li et al., 2020b). Therefore, to improve the malonic acid production, gene *glt-1* was incorporated into the *M. thermophila* SG214 strain, under the strong constitutive promoter of *gpdA* (MYCTH_2311855). After confirmation of the presence of the transgene by PCR analysis, the production of malonic acid by resultant strain SG317 was assayed. As shown in Figures 4B,C, the introduction of *glt-1* enabled faster glucose utilization and malonate production, while titer of malonic acid (42.5 mg/L) exhibited a slight increase compared with the parental strain SG214 (Figure 4B). These data suggested that glucose absorbability is not the key bottleneck for the current version of the cell factory, and other strategies are needed to increase the production of malonic acid.

Previous research on metabolic engineering of chemical products showed that effective export of target products is the key factor of efficient production of desirable products, and overexpression of exporters leads to the improved synthesis of organic acids, such as malic and fumaric acid (Chen et al., 2017). It was reported previously that *Anmae1*, a C4-dicarboxylate transporter from *A. niger*, has the capability to export malonic acid, and it has been introduced into *P. kudriavzevii* for increased malonate production (Dietrich et al., 2017). Disappointingly, when the gene *Anmae1* was overexpressed in the strain of SG214, under the control of the constitutive promoter of *pgk* (MYCTH_2316240), malonic acid production (41.3 mg/L) and glucose consumption rate by the resultant strain SG425 showed no significant increase, compared with the parent strain.

In order to improve the performance of cell factories for malonic acid production, more strategies of metabolic modification would be needed in the future, such as improvement of activity of key enzymes, disruption of branch points of the synthetic pathway, and enhancement of precursor pool size. In addition, intracellular high concentrations of malonate can competitively inhibit succinate dehydrogenase activity, which would lead to metabolic disturbance of host cells. Increasing host cell tolerance to malonate may be another strategy for the efficient production of malonic acid.

## Conclusion

In this study, we designed a novel artificial pathway for malonic acid biosynthesis with OAA as the substrate. The pathway was first tested *in vitro*. OAA can be converted to MSA by *a*-keto decarboxylase (Mdc) and then to malonic acid by the second enzyme (YneI). Then, using a cellulolytic fungus *M. thermophila* as the host, this novel pathway was functionally constructed *in vivo*. The present study provides a new potential option to produce malonic acid from glucose and even plant biomass in the future.

## Data Availability

The original contributions presented in the study are included in the article/Supplementary Material, further inquiries can be directed to the corresponding authors.
