# Supplementary material for: Designing and Constructing a Novel Artificial Pathway for Malonic Acid Production Biologically
Source: Front Bioeng Biotechnol. 2022 Jan 19;9:820507. doi: 10.3389/fbioe.2021.820507 (PMC8807515; doi:10.3389/fbioe.2021.820507)
Supplement: Supplementary file 1 [file Table1.DOCX]

Table S1. List of PCR primers used in this study

| **Name** | **Primer sequence (5’-3’)** | **Note** |
| --- | --- | --- |
| **Construction of vector in vitro for screening enzymes** | | |
| YneI-F | TAAGAAGGAGATATACATATGCATCATCACCATCACCATACCATTACTCCGGCAACCCA | Cloning of *YneI* |
| YneI-R | GGTGGTGGTGGTGCTCGAGTCAGACCCGGTCTTTCCACAC | Cloning of *YneI* |
| ARO10-F | TTAAGAAGGAGATATACATATGCATCATCACCATCACCATGCACCTGTTACAATTGAAAA | Cloning of *ARO10* |
| ARO10-R | GGTGGTGGTGGTGCTCGAGCTATTTTTTATTTCTTTTAAG | Cloning of *ARO10* |
| PDC-F | TTTAAGAAGGAGATATACATATGCATCATCACCATCACCATATGTCTGAAATTACTCTTG | Cloning of *PDC* |
| PDC-R | GGTGGTGGTGGTGCTCGAGTTATTGTTTGGCATTTGTAG | Cloning of *PDC* |
| Thi3-F | TTAAGAAGGAGATATACATATGCATCATCACCATCACCATAATTCTAGCTATACACAGAG | Cloning of *Thi3* |
| Thi3-R | GGTGGTGGTGGTGCTCGAGTCAGTATCCAACTTGATTTTT | Cloning of *Thi3* |
| Kivd-F | GAAGGAGATATACATATGCATCATCACCATCACCAT ATGTATACAGTAGGAGATTACCTATTAGAC | Cloning of *Kivd* |
| Kivd-R | GGTGGTGGTGGTGCTCGAGCTCGAGTTATGATTTATTTTGTTC | Cloning of *Kivd* |
| **Construction of overexpressing vector in *Myceliophthora thermophila*** | | |
| Ppgk-F | GCAGTTGGCTGACTTGAAGTAATCTCTGCAGATCTTGGCCATCGAGATCCACGAGC | Cloning of Ppgk |
| Ppgk-R | CTTGGTGATTCTACTGAGCAATTG | Cloning of Ppgk |
| Ppgk-YneI-F | GTCGCCAATTGCTCAGTAGAATCACCAAGATGACCATTACTCCGGCAACCCATGC | Cloning of *YneI* |
| Ppgk-YneI-R | GATGATTTCAGTAACGTTAAGTGGATCCGAATTCTCAGACCCGGTCTTTCCACAC | Cloning of *YneI* |
| Ppap-F | GCAGTTGGCTGACTTGAAGTAATCTCTGCAGATCTAATTCGCGGCCGCGGATCCAG | Cloning of Ppap |
| Ppap-R | GTTGACGGTTGTGTATGGAAG | Cloning of Ppap |
| Ppap-Mdc-F | GTACTTCACTCAATCTTCCATACACAACCGTCAACATGGGCCCTTTCCCTCTCTCGTC | Cloning of *Mdc* |
| Ppap-Mdc-R | GATTTCAGTAACGTTAAGTGGATCCGAATTCCTAACCGCACATGAAGTAGAATC | Cloning of *Mdc* |
| glt-1-F | TCGTTCTAGAATGGGTCTCTTCTCGAAAAAGT | Cloning of *glt-1* |
| glt-1-R | TGTTGTTAACCTAAACCTCTCCATGGCTTGAG | Cloning of *glt-1* |
| Anmae1-pgk-F | GTTCTGTCGCCAATTGCTCAGTAGAATCACCAAGACTAGTATGAACGTTGAAACGAG | Cloning of *Anmae1* |
| Anmae1-pgk-R | CTGTTTGATGATTTCAGTAACGTTAAGTGGATCCTCATTCAGACACATCCTCATCTTG | Cloning of *Anmae1* |
| **PCR analysis of gene overexpressing** | | |
| YneI-yz-F | CGACGTCACAGCTCCGAGAA | Detecing *YneI* overexpressing |
| YneI-yz-R | CTCGCTATTATTAGCCAGTT | Detecing *YneI* overexpressing |
| Mdc-yz-F | AACAGAAGCTAGACCCTTTG | Detecing *Mdc* overexpressing |
| Mdc-yz-R | AAGTGCTGGCCGATCAGCTTGA | Detecing *Mdc* overexpressing |
| glt-1-yz-F | CGACATCCGTTGAGCTCAA | Detecing *glt-1* overexpressing |
| glt-1-yz-R | CTGACCTTCTGGTTGTACAT | Detecing *glt-1* overexpressing |
| Anmae1-yz-F | AAGTACCTGCCATCAGGCCCA | Detecing *Anmae1* overexpressing |
| Anmae1-yz-R | AATCAGCCAACCATGTGTCG | Detecing *Anmae1* overexpressing |
