# Supplementary material for: Designing and Constructing a Novel Artificial Pathway for Malonic Acid Production Biologically
Source: Front Bioeng Biotechnol. 2022 Jan 19;9:820507. doi: 10.3389/fbioe.2021.820507 (PMC8807515; doi:10.3389/fbioe.2021.820507)
Supplement: Supplementary file 2 [file Table2.DOCX]

Table S2. Putative keto decarboxylases tested in this study

| **No.** | **GenBank** | **Original annotation** | **Species** |
| --- | --- | --- | --- |
| 1 | WP_143458661.1 (kivd) | keto acid decarboxylase | *Lactococcus lactis* |
| 2 | NP_010668.3 (ARO10) | phenylpyruvate decarboxylase | *Saccharomyces cerevisiae* |
| 3 | NC_001988.2 (PDC) | pyruvate decarboxylase | *Clostridium acetobutylicum* |
| 4 | NC_001139.9 (PDC6) | pyruvate decarboxylase | *Saccharomyces cerevisiae* |
| 5 | NP_010203.1 (THI3) | 2-oxoacid decarboxylase | *Saccharomyces cerevisiae* |
| 6 | XP_013934857.1 | Phenylpyruvate decarboxylase | *Ogataea parapolymorpha* |
| 7 | WP_034571939.1 | alpha-keto acid decarboxylase | *Carnobacterium divergens* |
| 8 | WP_069646548.1 | alpha-keto acid decarboxylase | *Enterococcus ureasiticus* |
| 9 | WP_119945901.1 | alpha-keto acid decarboxylase | *Brochothrix thermosphacta* |
| 10 | WP_208927415.1 | alpha-keto acid decarboxylase | *Enterococcus rotai* |
| 11 | ONH69602.1 | Transaminated amino acid decarboxylase | *Cyberlindnera fabianii* |
| 12 | XP_002492304.1 | Phenylpyruvate decarboxylase | *Komagataella phaffii* |
| 13 | QEU63000.1 | phenylpyruvate decarboxylase ARO10 | *Kluyveromyces lactis* |
| 14 | SMN19660.1 | Phenylpyruvate decarboxylase | *Kazachstania saulgeensis* |
| 15 | WP_012308362.1 | acetolactate synthase large subunit | *unclassified Synechococcus* |
| 16 | WP_106459190.1 | acetolactate synthase large subunit | *Aphanothece hegewaldii* |
| 17 | WP_083624637.1 | acetolactate synthase large subunit | *Planktothrix serta* |
| 18 | XP_829231.1 | 2-oxoglutarate dehydrogenase | *Trypanosoma brucei* |
| 19 | KNH06079.1 | hypothetical protein XU18_3016 | *Perkinsela sp.* |
| 20 | EPY42012.1 | 2-oxoglutarate dehydrogenase | *Angomonas deanei* |
| 21 | WP_011730279.1 | multifunctional oxoglutarate decarboxylase | *Mycolicibacterium smegmatis* |
| 22 | WP_005817136.1 | phosphonopyruvate decarboxylase | *Bacteroides fragilis* |
| 23 | WP_048583540.1 | phosphonopyruvate decarboxylase | *Streptomyces viridochromogenes* |
| 24 | Q54271.2 | Phosphonopyruvate decarboxylase | *Streptomyces hygroscopicus* |
| 25 | AEH16574.1 | Phosphonopyruvate decarboxylase | *Streptomyces hygroscopicus* |
| 26 | OWB82355.1 | lyase activity protein | *Candida boidinii* |
| 27 | KAF5117944.1 | hypothetical protein DV454_000837 | *Geotrichum candidum* |
| 28 | ODQ72087.1 | hypothetical protein | *Lipomyces starkeyi* |
| 29 | ONH78700.1 | Transaminated amino acid decarboxylase | *Saccharomyces cerevisiae* |
| 30 | VBB87765.1 | Phenylpyruvate decarboxylase | *Yarrowia lipolytica* |
| 31 | XP_002554859.1 | KLTH0F15510p | *Lachancea thermotolerans* |
| 32 | OXB45399.1 | hypothetical protein B1J91_A03102g | *Candida] glabrata* |
| 33 | XP_029322724.1 | uncharacterized protein | *Pichia kudriavzevii* |
| 34 | XP_003685687.1 | hypothetical protein TPHA_0E01600 | *Tetrapisispora phaffii* |
| 35 | WP_080222880.1 | alpha-keto acid decarboxylase | *Salmonella enterica* |
| 36 | OBT61556.1 | hypothetical protein VE03_08895 | *Pseudogymnoascus sp.* |
| 37 | XP_011121953.1 | hypothetical protein | *Arthrobotrys oligospora* |
| 38 | XP_031055525.1 | uncharacterized protein | *Fusarium odoratissimum* |
| 39 | CEJ61605.1 | hypothetical protein | *Penicillium brasilianum* |
| 40 | XP_022500140.1 | hypothetical protein | *Fonsecaea nubica* |
| 41 | THH27005.1 | hypothetical protein | *Antrodiella citrinella* |
| 42 | XP_025337651.1 | uncharacterized protein | *Candida] duobushaemulonis* |
| 43 | WP_164526149.1 | alpha-keto acid decarboxylase | *Proteus vulgaris* |
| 44 | XP_003868797.1 | hypothetical protein | *Candida orthopsilosis* |
| 45 | CDR42474.1 | CYFA0S09e04368g1_1 | *Cyberlindnera fabianii* |
